# Classification in Workhouses

**Published:** 1896-09-05

**Authors:** 


### Modern Sociology.

### CLASSIFICATION IN WORKHOUSES.
### II.—WHAT IT MIGHT ACCOMPLISH.

Last week we pointed out the urgent need which exists for the rearrangement of the existing system of administering poor law relief by Boards of Guardians. We showed why the time has come when it is essential to set up County Poor Law Boards and to abolish the existing Boards of Guardians, or to constitute them merely sub-committees of these new Boards. It has long been recognised by financiers who have studied the cost of the present poor law system, and the wastefulness which it engenders, that such a reconstruction as we have proposed would go a long way to solve the question of old age pensions.

The circular of the Local Government Board, dated July 31st, 1896, indicates certain reforms which a system of classification in woikhouses might bring about immediately. These include a provision by which those persons whose circumstances have compelled them to enter the workhouse, but who are known to be of good conduct and to have previously led moral and respectable lives, should be separated from those, who from their habits of speech or for other reasons, are likely to cause them discomfort. It is suggested that separate day-rooms for the inmates of each sex should be provided; that the workhouse arrangements should be relaxed so as to give special facilities to the friends of the deserving poor to visit them; and that such inmates should be allowed more than ordinary liberty to visit their friends, to attend their own place of worship on Sunday, and for other purposes. Arrangements might also be made for providing every such inmate with a separate room or cubicle to sleep in. Girls of blameless character, or who have been admitted to the workhouse for their first confinement, should be separated from women whose previous life has been such that their influence must prejudicially affect those who are associated with them in laundry work, the nurseries, or in other ways, by day or by night.

In our previous article we gave our reasons for believing that the foregoing advantages can only be secured by placing all the poor law buildings and lands in a given area under a County Poor Law Board. Unless this is done classification can never be adequately carried out, and the advantages which the Local Government Board rightly claim for the honest and respectable poor can never be provided effectually or at a reasonable cost. The existing system entails a considerable waste of money, for within the same county some workhouses are crowded whilst others are comparatively empty, and some of the buildings are sufficiently modern to provide reasonable accommodation, whilst others are so bad or ill-adapted for the purposes of classification as to make any attempt to enforce it a palpable failure from the outset. On moral grounds alone the present system is a failure, for no matter how intelligent and careful the administration may be, it is found in practice that the workhouses in large cities are successfully used by thieves and others as recruiting grounds for their immediate purposes. Steps have been and are being taken to remove pauper children from the baneful influences of the existing workhouse system, but so long as district schools are persisted in without the protection of an adequate age clause which will render it impossible for the parents to remove the children until they have thoroughly mastered a trade and attained an age when they can maintain themselves respectably by their own earnings, the circumstances of the pauper child must tend to keep it a pauper throughout the whole term of its natural life.

We hope, therefore, that Mr. Chaplin will signalise his term of office as President of the Local Government Board by moving the present Government, which is instinct with a desire to improve the social condition of the people, to introduce a Bill setting up County Poor Law Boards on a plan, and for the reasons already stated. If this were done it would prepare the way for the introduction of a practical measure, whereby old age pensions might easily be provided for the deserving poor of both sexes. The institution of County Poor Law Boards and the adequate system of classification in workhouses which would follow their formation as a natural consequence, would at once simplify the pension question by reducing the number of poor persons to be thus provided for to reasonable proportions. A large number of the deserving poor have few friends and no adequate homes of their own, and these would certainly prefer to accept a provision for their maintenance and accommodation in the reformed workhouses where their associates would be confined to respectable, honest, and moral persons. Not only would the accommodation and surroundings of the reformed workhouse tend to make inmates such comfortable and happy, but the increased liberty and the opportunities afforded for healthy outdoor occupation in the garden and grounds, or in the workroom and establishment, would possess greater attractions for the classes in question than any system of pensions could reasonably be expected to offer.

It follows that the number of persons who would be eligible for out-pensions would be largely reduced, while the increased economy and the consequent large reduction in expenditure, which must result from the consolidation of the existing boards of guardians into County Poor Law Boards, would place a considerable annual sum at the disposal of the Government, which might be applied to purposes of old-age pensions without any increase in the existing rates. We have long realised the possibility of securing such a result in the manner here proposed, and the more the figures are studied the more practical will our scheme be found to be. We have only space left to advance one proof of the soundness of the faith within us in regard to this question. It consists in the knowledge that it costs more than twice as much to maintain a pauper in the metropolis that it does to maintain him in any other place in England, whilst the contract prices paid for various articles supplied to Boards of Guardians, as given in the reports of the Local Government Board, indicate clearly the gross peculation or the hideous wastefulness of Boards of Guardians. Here, indeed, is a gold mine from which Lord Rothschild's Committee might extract hundreds of thousands of pounds with great advantage to the whole community.

